# Do health care institutions value research? A mixed methods study of barriers and facilitators to methodological rigor in pediatric randomized trials

**DOI:** 10.1186/1471-2288-12-158

**Published:** 2012-10-18

**Authors:** Michele P Hamm, Shannon D Scott, Terry P Klassen, David Moher, Lisa Hartling

**Affiliations:** 1 Alberta Research Centre for Health Evidence, Department of Pediatrics, Faculty of Medicine and Dentistry, University of Alberta, Edmonton, AB, Canada; 2 Faculty of Nursing, University of Alberta, Edmonton, AB, Canada; 3 Department of Pediatrics, Faculty of Medicine and Dentistry, University of Alberta, Edmonton, AB, Canada; 4 Department of Pediatrics and Child Health, Faculty of Medicine, University of Manitoba, Winnipeg, MB, Canada; 5 Ottawa Hospital Research Institute, Ottawa, ON, Canada; 6 Department of Pediatrics, University of Alberta, ECHA 4-482B, 11405-87 Avenue, Edmonton, AB, T5G 1C9, Canada

**Keywords:** Clinical trials as topic, Risk of bias, Pediatrics, Mixed methods

## Abstract

**Background:**

Pediatric randomized controlled trials (RCTs) are susceptible to a high risk of bias. We examined the barriers and facilitators that pediatric trialists face in the design and conduct of unbiased trials.

**Methods:**

We used a mixed methods design, with semi-structured interviews building upon the results of a quantitative survey. We surveyed Canadian (n=253) and international (n=600) pediatric trialists regarding their knowledge and awareness of bias and their perceived barriers and facilitators in conducting clinical trials. We then interviewed 13 participants from different subspecialties and geographic locations to gain a more detailed description of how their experiences and attitudes towards research interacted with trial design and conduct.

**Results:**

The survey response rate was 23.0% (186/807). 68.1% of respondents agreed that bias is a problem in pediatric RCTs and 72.0% felt that there is sufficient evidence to support changing some aspects of how trials are conducted. Knowledge related to bias was variable, with inconsistent awareness of study design features that may introduce bias into a study. Interview participants highlighted a lack of formal training in research methods, a negative research culture, and the pragmatics of trial conduct as barriers. Facilitators included contact with knowledgeable and supportive colleagues and infrastructure for research.

**Conclusions:**

A lack of awareness of bias and negative attitudes towards research present significant barriers in terms of conducting methodologically rigorous pediatric RCTs. Knowledge translation efforts must focus on these issues to ensure the relevance and validity of trial results.

## Background

“We as an institution, as a profession, don’t actually sell research as being an important thing that we do in hospitals. And it should be.”

There is a growing body of literature documenting the methodological limitations of published randomized controlled trials (RCTs) in pediatrics [1-7]. Of particular concern is the evidence that RCTs in child health are susceptible to a high risk of bias, increasing the likelihood that reported treatment benefits and/or harms are being exaggerated [8-10]. In order to ensure clinical relevance and to prevent unnecessary and wasteful research, it is crucial that measures are taken to maximize the internal validity of studies that are conducted [11,12]. The global investment in research is enormous, with funding of $100 billion annually [11], plus the time and effort committed by the researchers, clinicians, and children and families. When participants agree to take part in a trial, they expect that the study will be conducted and reported to the highest standard to accurately answer the research question. When this expectation is met, trial results are important in providing children with the best possible treatment; however, when biased research is conducted instead, research dollars and professionals’ time are wasted, and the children’s contributions are unavailing.

Evidence describing the negative impact of bias on RCTs and how to minimize it is available [13-22], as is research on a number of specific challenges inherent in conducting RCTs in pediatrics [23-27], such as recruitment and consent procedures. However, the research-practice gap regarding methodological rigor in this population has not yet been addressed.

As the first step in the development of a knowledge translation strategy to address the reduction of bias in pediatric RCTs, the objective of this study was to determine and describe the barriers and facilitators that pediatric trialists face in the design and conduct of unbiased trials, with an emphasis on the Canadian context. Quantitative survey and qualitative interview data were collected to gain a broad perspective of the problem and researchers’ experiences.

## Methods

### Design

We used an explanatory mixed methods design, with semi-structured interviews building upon the results of a quantitative survey. We connected data from the two phases to provide detailed descriptions of the barriers and facilitators pediatric trialists face in designing and conducting studies with high internal validity [28]. We obtained ethical approval from the Health Research Ethics Board at the University of Alberta.

### Data collection

#### Quantitative: survey

We sent an Internet-based survey (SurveyMonkey) to a sample of Canadian and international pediatric trialists between September 2010 and February 2011. We searched the Cochrane Central Register of Controlled Trials for pediatric RCTs published in 2008 and 2009 and identified 7,535 articles. Corresponding authors of all relevant trials identified using a Canadian-specific search filter (n=90), and a geographically representative sample of 600 international trialists chosen from a randomly ordered list were invited to participate in the survey. We sent an invitation e-mail with a link to the survey and two reminder e-mails separated by two week intervals. The questionnaire was informed by the Cochrane Collaboration’s Risk of Bias tool [29], the BARRIERS Scale [30], and the framework proposed by Cabana et al. [31], described in Table 1.

**Table 1:** Tools used for survey development

**Tool**	**Description**
Risk of Bias tool	Used to assess the internal validity of RCTs. It is comprised of seven domains supported by empirical evidence: sequence generation, allocation concealment, blinding of participants and personnel, blinding of outcome assessors, incomplete outcome data, selective outcome reporting, and “other” sources of bias [29].
BARRIERS Scale	Widely used to identify general barriers to research utilization, particularly in nursing. Barriers are categorized into factors related to the individual, setting, research, and presentation [30,32].
Cabana framework	Developed by Cabana et al. [31] as part of an evaluation of barriers to physicians’ adoption of clinical practice guidelines. This framework includes 10 major factors, grouped into categories related to knowledge, attitudes, and behaviour.

Twelve methodologists and clinicians evaluated the questionnaire for appropriateness and accuracy of content and 5 national and international pediatric trialists completed pilot testing for clarity and ease of use; we made revisions based on their feedback. The survey included 23 questions and pilot testing indicated that it would take approximately 15 minutes to complete. We developed items to determine: 1) researcher knowledge and awareness of bias; and 2) perceived barriers and facilitators in conducting clinical trials.

Due to a low response rate using the sample described above (154/644; 23.9%), we expanded the survey population to recruit participants from the membership of the Maternal Infant Child and Youth Research Network (MICYRN), a Canadian network linking investigators from 17 academic health centres involved in pediatric clinical research. We identified potential respondents through a publicly available network inventory maintained by MICYRN (http://www.micyrn.ca/Networks.html) and invited all individuals listed as network contacts via email to participate in both the survey and the interview portion of the study (n=163). The survey included an item asking whether respondents would be willing to be contacted for an interview, and if so, to provide their name and preferred means of initial contact. As a result of problems with access to SurveyMonkey, we administered this wave via REDCap, an alternate secure, online application for managing surveys.

#### Qualitative: interviews

Due to low participation rates from survey respondents, we augmented recruitment with members of MICYRN with trial experience and referrals from participants and established pediatric trialists. We used purposive sampling based upon pediatric subspecialty and geographic location, aiming to reach saturation, which typically occurs around 12 participants [33]. Interviews followed a semi-structured format built upon the results of the survey and were focused on participants’ experiences and attitudes towards conducting pediatric research and how these interacted with the appropriate design and conduct of methodologically sound trials. Each participant was sent an electronic consent form that they signed and returned via fax or email prior to the conduct of the interview. Interviews were 30 to 60 minutes and conducted by telephone by the lead author between April and July 2011. All interviews were recorded and transcribed verbatim.

#### Data analysis

We analyzed survey data descriptively, using means and standard deviations, medians and interquartile ranges (IQR), or proportions where appropriate. We used content analysis to code interviews, identifying categories in the data and patterns in beliefs and values that could help explain the potential for bias in pediatric RCTs [34]. Coding was conducted by the lead author in consultation with the rest of the study team. We conducted qualitative data collection and analysis concurrently, following an iterative process. We integrated the survey and interview data at the data interpretation phase [35], using the method of connecting data [28]. We used Stata and NVivo to manage quantitative and qualitative data, respectively.

## Results

The survey response rate was 23.0% (186/807) and 13 interviews were conducted. Characteristics of the survey and interview participants are described in Tables 2 and 3 and Additional file 1. Survey results were similar across geographic boundaries and were therefore combined and used as a whole to inform a detailed examination of how barriers and facilitators manifest in Canadian research. Results are presented according to their classification as individual, institutional, or policy level factors. Themes are outlined in Table 4.

**Table 2:** Demographics of survey population

**Variable**	**n (%)**
Total returned surveys	186/807 (23.0)
Undeliverable surveys	46/853 (5.4)
Professional time spent on research-related activities	
0%–25%	34 (18.3)
26%–50%	28 (15.1)
51%–75%	48 (25.8)
76%–100%	38 (20.4)
No response	38 (20.4)
Involvement in RCTs – median number of trials (IQR)	
As a principal investigator	3 (1–5)
As a member of the study team	5 (2–10)
Discipline trained in*	
Medicine	83 (44.6)
Research	69 (37.1)
Psychology	17 (9.1)
Allied healthcare	13 (7.0)
Nursing	11 (5.9)
Other	9 (4.8)
No response	38 (20.4)
Pediatric subspecialty	
Public health	16 (8.6)
Developmental, psychosocial, and learning problems	14 (7.5)
Mental health or psychiatry	13 (7.0)
Neonatology	11 (5.9)
Endocrinology and nutrition	10 (5.4)
Emergency medicine or critical care	9 (4.8)
Infectious diseases	9 (4.8)
Hematology or oncology	7 (3.8)
Oral health	6 (3.2)
Allergy and immunology	5 (2.7)
Anesthesia	5 (2.7)
General pediatrics or family medicine	5 (2.7)
Other	47 (25.2)
No response	29 (15.6)
Geographic region of corresponding author	
Asia	10 (5.4)
Australia and New Zealand	12 (6.5)
Canada	47 (25.2)
Europe	25 (13.4)
South America	3 (1.6)
USA	46 (24.7)
No response	43 (23.1)
Setting of employment*	
University or academic centre	124 (66.7)
Hospital	48 (25.8)
Solo practice	4 (2.2)
Group practice	4 (2.2)
Industry	4 (2.2)
Other	7 (3.8)
No response	39 (21.0)

*More than one selection possible.

Further details on collapsed categories are available from the authors.

**Table 3:** Characteristics of interview participants

**Variable**	**n (%)**
Total interviews	13 (100)
Source of recruitment	
Survey	3 (23.1)
MICYRN	3 (23.1)
Referral from participants/established trialists	7 (53.8)
Discipline(s) trained in	
Medicine	7 (53.8)
Research	2 (15.4)
Medicine and Research	4 (30.8)
Pediatric subspecialty	
Anesthesiology	1 (7.7)
Clinical epidemiology	1 (7.7)
Critical care	2 (15.4)
Emergency medicine	3 (23.1)
Infectious disease	1 (7.7)
Neonatology	1 (7.7)
Neurology	1 (7.7)
Oncology	1 (7.7)
Psychology	1 (7.7)
Rheumatology	1 (7.7)
Geographic region of participant	
Alberta	2 (15.4)
British Columbia	2 (15.4)
Ontario	6 (46.2)
Quebec	1 (7.7)
USA	2 (15.4)

**Table 4:** Interview themes and relevance to risk of bias

**Category**	**Theme**	**Relevance to risk of bias**
*Barriers*		
Individual	Knowledge	- Little formal training in research methods, therefore bias is likely due to a lack of knowledge of how it is introduced.
Institutional	Clinical care vs. clinical research	- Decisions made clinically rather than per the trial design can lead to protocol deviations, e.g. interference with randomization sequence.
	Culture	- Research is often viewed negatively in the clinical setting, leading to little value placed on following the trial protocol when it deviates from usual care.
	Logistics	- Demands on time and space can put research at a low priority and tasks may not be done according to protocol, e.g. ensuring safeguards are in place to maintain blinding.
Policy	Administration	- Budget constraints can limit hiring external methodological expertise if necessary; ethics requirements for methodology are inconsistent, leaving protocols subject to change.
	Pediatric-specific challenges	- Blinding parents; investigators are less willing to inconvenience families with strict protocols; fewer trials has meant less competition for developing the best methodology.
*Facilitators*		
Individual	Ownership	- The trial will be more successful when the investigators take responsibility for generating support and ensuring rigor.
Institutional	Acceptance	- Researcher understanding of the clinical setting facilitates the acceptance of research methods by the practitioners.
	Cohesive study team	- Consulting experienced trialists and methodologists contributes to a more rigorous and well thought out study, in terms of both validity and feasibility.
	Infrastructure	- Protected research time and dedicated research staff facilitate trial design and conduct.
	Verification	- Checks on the science facilitate high quality, e.g., reliable review processes and guidance from trusted third parties.

### Individual factors

Survey findings indicated that 68.1% of respondents agree that bias is a problem in pediatric RCTs and 72.0% reported that they felt there was sufficient evidence to support the need to change some aspects of how RCTs are conducted. However, knowledge of bias among respondents was variable. There was no consistency in responses to questions which asked the respondent to rate the degree to which they agreed that a study design factor would introduce bias into a study. Identification of specific biases was strongest for sequence generation, blinding, and selective outcome reporting, while there was more uncertainty surrounding identification of problems with allocation concealment, incomplete outcome data, and “other sources of bias” (see Additional file 2). Despite this range of awareness of issues relevant to bias, 94.2% of respondents felt confident in their ability to evaluate the quality of published trials.

The interviews highlighted two important themes regarding barriers and facilitators at the individual level: knowledge and training regarding research methods, and engagement or ownership in the research process (Table 5). While most survey respondents indicated that bias is a problem, the interview data suggested that trialists often do not have the knowledge to first, recognize, and second, address bias in their studies. They often mentioned a lack of formal training, instead relying on skills learned on the job.

**Table 5:** Perspectives on individual-level factors

*Barriers*	
02	Probably we don’t look at, we don’t know all the bias that can be, that can happen in a trial because we don’t check, we don’t believe there’s bias. We may miss some, we may forget some, and then do not report the bias because we don’t know it exists.
06	I’ve kind of learned on the job, which is why I’m not fully confident that I have all the skills.
07	Because there’s almost zero research training in the clinical curriculum for most clinicians these days. Like there’s almost nothing in the med school program, there’s almost nothing in the rehab program – there really needs to be somebody on the protocol who’s got a little bit more training.
10	Well you know it’s often when people go to write up a protocol, either they’re not totally aware of how this whole bias thing works and to them, you know the fact that you randomize people by the day of the week they present, that sounds good enough.
*Facilitators*	
04	So you really have to take the time to engage people and be the one that’s proactive, engaging them. Because they’re busy, they might not even know what your study is unless you’re the change agent that really goes out there and talks to them about it and gets them motivated about why you think it’s important.

Conversely, a sense of ownership can contribute to a rigorous study design. Actively taking responsibility for the direction of the trial was seen as an opportunity for the investigators to generate enthusiasm, gain support, and educate colleagues about the rationale for rigorous methodology and how it impacts the ability to accurately answer the research question.

"“Listen to what [your colleagues] need to execute the study so that when you develop your protocol, you’ve built that into the approach. Or, if you couldn’t, you’ve at least had that dialogue with them about how scientifically you can’t be as flexible as might be ideal… so that they at least understand the rationale.”"

### Institutional factors

While 93.0% of survey respondents demonstrated an interest in learning about and staying current with literature describing and analyzing research methods, only 50.3% felt that they were able to do so due to other constraints. Logistical issues such as meeting institutional requirements (29.2%) and having sufficient staff (30.4%) were identified as challenges, while access to knowledgeable colleagues (92.8%) was identified as the most significant facilitator (see Additional file 2).

Consistently, interview respondents felt that environmental factors within their institutions were not conducive to research, often as the result of perceptions of research (Table 6). They reported the underlying culture to be overwhelmingly negative towards research at all levels, with resistance from trainees, nurses, physicians, administrators, and the pharmaceutical industry. While respondents felt that the products of research tend to be valued once they are demonstrated to improve practice, they stated that there is little appreciation of the methods necessary to achieve that goal. Logistically, respondents felt that having staff dedicated to research would improve this situation, as research procedures would then not consistently be placed at the lowest priority. Recognition of the division between the paradigms of clinical care (e.g., protecting the interests and comfort of patients) and clinical research (e.g., maintaining clinical equipoise) was also identified as a challenge. Clinical investigators often have difficulty with this distinction, which allows for the possibility of compromised trial protocols.

**Table 6:** Perspectives on institution-level factors

*Barriers*	
09	I think that other people view [research] as kind of a thorn in their side. It’s something they play along with if they have to and the division head tells them they have to.
09	You work separately or in parallel and not necessarily the team as much, and I think that’s part of the challenge. You view the study as important, they view the results as important, but they don’t want to go through the pain of finding out the results because it impacts on what they do clinically.
03	Where we get into the biggest problems is if we take a person who’s very knowledgeable and very confident in how to care for patients with [*disease*], so they’re experts and masters in clinical care, and they just assume that that carries over into being an expert in clinical research.
*Facilitators*	
01	So because we get donated funds, a fairly large amount of donated funds proportionally speaking in [*disease*], we’re able to support the personnel to perform the trials.
06	We have the help of the research institute and you can have a person for any kind of question or any kind of design that can help, and we have access to those kinds of resources.
09	I think the fact that [*research network*] is there enables you to think of multi-centre RCTs, whereas if it wasn’t there, you’d kind of have to go and find things from scratch. But by existing, it brings people together with shared interests and I think that that is a huge asset when it comes to even the thought of designing a multi-centre RCT. It’s like you want to design one for [*research network*].
07	[*Research institute*] has a great model where they require any grant that’s going out for external funding to be reviewed by three people from inside the institution.

"“I think a lot of investigators really have a hard time separating what decision they would make clinically from what decision they would make as part of a trial… because the feeling is I want to be convenient to the family, and I really know this stuff because I’m an expert in this clinical area, and I don’t think they realize that there’s a pretty clear demarcation between what you do in clinical care and what you do as research.”"

The degree to which institutional barriers were perceived as a threat to research varied according to the size of the site of the respondent. A clear distinction was noted between larger research-intensive institutions and other sites in which researchers struggled due to a lack of infrastructure. Trialists from the former viewed the conduct of research much more positively and generally felt that they had the necessary resources available to conduct rigorous trials, while those from the latter reported greater levels of difficulty positioning their research as an important part of the clinical landscape.

"“Space, resources, and training for research assistants, research managers, graduate students… there’s all sorts of hurdles and headaches around those things that I think most established clinical research programs… already know how to make the system work.”"

Similar to the survey results, cohesive study teams with positive working relationships were reported as the most significant facilitator to conducting rigorous trials. At the institutional level, this often included combining the expertise of experienced trialists, methodologists, and the staff that would be responsible for implementing the trial; however, this integration was more common at sites with more support for research. Positive relationships were also mentioned in the context of subspecialties, with productive research networks across sites enabling researchers to benefit from a collective expertise, as well as facilitating study-specific elements such as the conduct of multi-centre trials. A final facilitator, which was more prominent in certain institutions than others, was a reliable internal review process. Respondents viewed this as a major asset when it was available, but many felt that existing processes were fragmented and inconsistent.

### Policy factors

All interview respondents felt a lack of incentive to conduct pediatric trials. Difficulty in securing funding was frequently mentioned, but beyond that, participants reported that it is challenging to justify including financial support for a methodologist in grant application budgets in an environment where the funds awarded are anticipated to be less than requested. In this context, there is less assurance that study teams can include the necessary expertise. Meeting the requirements of several research ethics boards also presents a challenge, particularly with the preponderance of multi-centre trials in pediatrics. With separate ethics approval processes at each institution, the process can be lengthy and protocols are oftentimes changed to meet the inconsistent requests of the individual review panels (Table 7).

**Table 7:** Perspectives on policy-level factors

*Barriers*	
13	We all tend to want to make the budget as small as we can to increase our chances to actually get it funded and the reality is that some trials really require the full-time effort of somebody who’s got a lot of experience, and therefore comes with a price tag. And it can be hard to make the argument to ensure that you’ve got funding, right? So I think that’s where you start cutting other corners, and you don’t have the data quality, and at the end of the day, you maybe don’t have the rigorous, homerun kind of trial that you had envisioned.
02	*(regarding ethics review at multiple sites)* Most of the problem is to ask for revisions and they are not consistent one between the others. So you can have a question in one and the other one… wants a different answer.

### Specific biases and pediatric-specific challenges

Addressing specific biases, survey and interview respondents reported challenges with blinding most frequently, which included the cost of providing a placebo, difficulties in blinding non-pharmacological interventions, and blinding all relevant parties, including parents. Other issues that were mentioned included difficulty getting adequate follow up in settings without an established clinician-patient relationship, parental resistance to randomization, and group imbalances due to small sample size.

## Discussion

Bias is a recognized concern among pediatric trialists; however they may be lacking the knowledge, willingness, or resources to properly address it. Internal validity did not emerge as a primary concern in the analyses, being overshadowed by issues related to the pragmatics of running a trial and the generalizability of the results. While these issues warrant a great deal of consideration, it is crucial that studies start out being methodologically rigorous as this is a prerequisite for generalizability.

The major barriers to minimizing risk of bias in trials were related to awareness and environment. With little emphasis on research methodology in clinical curricula, many investigators are not adequately prepared to design trials with high levels of internal validity or to recognize and attend to issues as they arise. The existing *ad hoc* training system very likely contributes to an emphasis on certain areas and a deficit in others, as demonstrated by the disproportionate focus by respondents on issues related to external validity (i.e., generalizability of study results), despite being questioned on issues relevant to internal validity (i.e., avoiding bias through methodologically rigorous design). Additionally, the predominantly negative attitudes surrounding the research process reinforce the acceptance of sub-optimal RCTs. While research findings may be valued, more effort is required to ensure that the importance of high quality research is recognized at all stages, and by all stakeholders. Pediatric oncology is often cited as a model in developing an environment that fosters research. Available infrastructure and consistency in study protocols has resulted in the successful integration of research and clinical care leading to marked improvements in survival and other outcomes [24,36,37]. By embracing research as a critical component of providing best care, rather than viewing it as an imposition, investigators and clinicians in oncology have shown that setting a standard for conducting rigorous trials is an achievable goal with tangible benefits and impressive health outcomes.

Positive relationships that support the development of an interest in research are particularly relevant in an environment in which most training is dependent on mentorship and reinforcement from experienced trialists. Within this context, clinician-scientists have a key role in bridging the gap between the worlds of research and clinical practice. Combining knowledge of proper methodology with an appreciation for the demands of the clinical setting will increase the likelihood of producing both valid and realistic trials. Research networks such as the Canadian Critical Care Trials Group (CCCTG) and Pediatric Emergency Research Canada (PERC) have been quite successful in using this strategy, facilitating high quality trials by promoting a positive culture of research, providing access to individuals with expertise, and offering support and collegiality [38,39].

With a solid evidence base demonstrating the gaps in methodological quality in pediatric RCTs [1-10], the research agenda must now focus on knowledge translation. Using barriers and facilitators identified by the target end-users, it will be important to develop tailored strategies to overcome the gap between what is known about methodological processes and how trials are designed and conducted in practice. This is one of the stated aims of StaR Child Health, an international initiative dedicated to improving the quality of pediatric clinical research [40].

### Strengths and limitations

An advantage of this study is that it combines the breadth of survey responses with the depth of interview responses, allowing for a detailed picture of the barriers and facilitators pediatric trialists face in the conduct of methodologically rigorous trials. While the response rate to the survey was low, bringing into question the representativeness of the sample, it was in line with evidence that both electronic surveys [41] and physician surveys [42] are associated with low responses. However, respondents represented a wide range of pediatric specialties, training backgrounds, and geographies, helping to give shape to the subsequent interviews. While they may have represented researchers with a higher level of interest in methodology, the survey responses were used to form the interviews, in which researchers with trial experience were of interest so as to be able to account for the barriers and facilitators in pediatric research with first-hand knowledge. The recruitment of additional survey participants from the membership of MICYRN slightly changed the balance of geographical representation; however the Canadian context was weighted heavily throughout the study, therefore our emphasis was unchanged. The response rate to requests for interview participation was also low, but with our expanded recruitment strategy we were able to achieve saturation. The interviews emphasized the Canadian context and therefore can be used to inform future developments within the national health care and research framework, as well as provide considerations relevant to other settings.

## Conclusions

Clinical research is inherently challenging, but these results can be used to focus efforts on improving the validity of trials that are conducted. The evidence is clear that improvement is necessary in pediatric RCTs and a substantial body of knowledge has accumulated around how to minimize bias. Before trials can improve, though, awareness of bias and attitudes towards research must be addressed. Research must be reframed as a valuable component of health care education, practice, and decision-making.

## Abbreviations

CCCTG: Canadian Critical Care Trials Group; IQR: Interquartile range; MICYRN: Maternal Infant Child and Youth Research Network; PERC: Pediatric Emergency Research Canada; RCT: Randomized controlled trial.

## Competing interests

The authors declare that they have no competing interests.

## Authors’ contributions

MPH collected and analyzed the data, drafted the manuscript, and integrated critical feedback from the other authors. All of the authors were involved in the development of the proposal and interpretation of the data. All of the authors provided feedback on the revisions to the manuscript and approved the final version submitted for publication.

## Pre-publication history

The pre-publication history for this paper can be accessed here:

http://www.biomedcentral.com/1471-2288/12/158/prepub

## Supplementary Material

Additional file 1 **a: Demographics of survey population – original sample.** b: Demographics of survey population – MICYRN sample. Click here for file

Additional file 2 Survey results. Click here for file
