# Supplementary material for: Do health care institutions value research? A mixed methods study of barriers and facilitators to methodological rigor in pediatric randomized trials
Source: BMC Med Res Methodol. 2012 Oct 18;12:158. doi: 10.1186/1471-2288-12-158 (PMC3503580; doi:10.1186/1471-2288-12-158)
Supplement: Additional file 1 — a: Demographics of survey population – original sample. b: Demographics of survey population – MICYRN sample. [file 1471-2288-12-158-S1.doc]

**Additional File 1a. Demographics of survey population – original sample**

| **Variable** | **n (%)** |
| --- | --- |
| Total returned surveys  Undeliverable surveys | 154/644 (23.9)  46/690 (6.7) |
| Professional time spent on research-related activities  0%–25%  26%–50%  51%–75%  76%–100%  No response | 28 (18.2)  26 (16.9)  35 (22.7)  33 (21.4)  32 (20.8) |
| Involvement in RCTs – median number of trials (IQR)  As a principal investigator  As a member of the study team | 3 (2-5)  4 (2-10) |
| Discipline trained in*  Medicine  Research  Psychology  Allied healthcare  Nursing  Other  No response | 62 (40.3)  59 (38.3)  17 (11.0)  11 (7.1)  10 (6.5)  9 (5.8)  32 (20.8) |
| Pediatric subspecialty  Public health  Developmental, psychosocial, and learning problems  Mental health or psychiatry  Neonatology  Endocrinology and nutrition  Emergency medicine or critical care  Infectious diseases  Hematology or oncology  Oral health  Allergy and immunology  Anesthesia  General pediatrics or family medicine  Other  No response | 16 (10.4)  9 (5.8)  12 (7.8)  8 (5.2)  8 (5.2)  7 (4.5)  9 (5.8)  6 (3.9)  6 (3.9)  5 (3.2)  5 (3.2)  3 (1.9)  39 (25.3)  23 (14.9) |
| Geographic region of corresponding author  Asia  Australia and New Zealand  Canada  Europe  South America  USA  No response | 10 (6.5)  12 (7.8)  24 (15.6)  25 (16.2)  3 (1.9)  43 (27.9)  37 (24.0) |
| Setting of employment*  University or academic centre  Hospital  Solo practice  Group practice  Industry  Other  No response | 98 (63.6)  36 (23.4)  4 (2.6)  2 (1.3)  4 (2.6)  6 (3.9)  33 (21.4) |

*More than one selection possible.

Further details on collapsed categories are available from the authors.

**Additional File 1b. Demographics of survey population – MICYRN sample**

| **Variable** | **n (%)** |
| --- | --- |
| Total returned surveys | 32/163 (19.6) |
| Professional time spent on research-related activities  0%–25%  26%–50%  51%–75%  76%–100%  No response | 6 (18.8)  2 (6.3)  13 (40.6)  5 (15.6)  6 (18.8) |
| Involvement in RCTs – median number of trials (IQR)  As a principal investigator  As a member of the study team | 3 (1-6)  10 (4-15) |
| Discipline trained in*  Medicine  Research  Psychology  Allied healthcare  Nursing  Other  No response | 21 (65.6)  10 (31.3)  0 (0.0)  2 (6.3)  1 (3.1)  0 (0.0)  6 (18.8) |
| Pediatric subspecialty  Public health  Developmental, psychosocial, and learning problems  Mental health or psychiatry  Neonatology  Endocrinology and nutrition  Emergency medicine or critical care  Infectious diseases  Hematology or oncology  Oral health  Allergy and immunology  Anesthesia  General pediatrics or family medicine  Other  No response | 0 (0.0)  5 (15.6)  1 (3.1)  3 (9.4)  2 (6.3)  2 (6.3)  0 (0.0)  1 (3.1)  0 (0.0)  0 (0.0)  0 (0.0)  2 (6.3)  8 (25.0)  6 (18.8) |
| Geographic region of corresponding author  Canada  USA  No response | 23 (71.9)  3 (9.4)  6 (18.8) |
| Setting of employment*  University or academic centre  Hospital  Solo practice  Group practice  Industry  Other  No response | 26 (81.3)  12 (37.5)  0 (0.0)  2 (6.3)  0 (0.0)  1 (3.1)  6 (18.8) |

*More than one selection possible.

Further details on collapsed categories are available from the authors.
