# Supplementary material for: Do health care institutions value research? A mixed methods study of barriers and facilitators to methodological rigor in pediatric randomized trials
Source: BMC Med Res Methodol. 2012 Oct 18;12:158. doi: 10.1186/1471-2288-12-158 (PMC3503580; doi:10.1186/1471-2288-12-158)
Supplement: Additional file 2 — Survey results. [file 1471-2288-12-158-S2.doc]

**Additional file 2. Survey results**

**Individual-level factors**

| **Item** | **Disagree**  **n (%)** | **Neutral**  **n (%)** | **Agree**  **n (%)** | **Don’t Know**  **n (%)** | **Total**  **n** |
| --- | --- | --- | --- | --- | --- |
| Bias, or systematic error, is a problem in pediatric trials | 29 (15.7) | 24 (13.0) | **126 (68.1)** | 6 (3.2) | 185 |
| In general, researchers consider a trial’s potential for bias when they are making clinical recommendations based on the study’s results | 32 (17.5) | 18 (9.8) | **124 (67.8)** | 9 (4.9) | 183 |
| Any trial conducted is better than no trial at all | **130 (70.7)** | 7 (3.8) | 46 (25.0) | 1 (0.5) | 184 |
| I am aware of published research on bias in randomized controlled trials | 16 (10.1) | 8 (5.1) | **132 (83.5)** | 2 (1.3) | 158 |
| I keep up with published research on bias in randomized controlled trials | 52 (33.1) | 25 (15.9) | **79 (50.3)** | 1 (0.6) | 157 |
| I am interested in staying current with literature describing and analyzing research methods | 4 (2.5) | 7 (4.5) | **146 (93.0)** | - | 157 |
| I find the volume of published literature on research methods overwhelming and have trouble keeping up with this literature | 28 (17.8) | 26 (16.6) | **99 (63.1)** | 4 (2.5) | 157 |
| I think there is evidence that changes are needed to some aspects of how randomized controlled trials are conducted | 7 (4.5) | 31 (19.7) | **113 (72.0)** | 6 (3.8) | 157 |
| I feel that research on bias is relevant to my work | 2 (1.3) | 5 (3.2) | **150 (95.5)** | - | 157 |
| I am receptive to new ideas that may lead to changes in how I conduct my research | - | 1 (0.6) | **154 (98.7)** | 1 (0.6) | 156 |
| Minimizing bias is an issue I am conscious of when conducting my research | 2 (1.1) | 5 (2.7) | **176 (95.7)** | 1 (0.5) | 184 |
| I am confident in my ability to evaluate the quality of published trials | 4 (2.6) | 5 (3.2) | **145 (94.2)** | - | 154 |
| How confident are you in your understanding of what bias is? | **mean (SD):** 5.5 (1.2) [7 point scale] | | | | |
| How likely is it that you will consider risk of bias in the next trial in which you are involved? | **mean (SD):** 6.2 (1.3) [7 point scale] | | | | |
| How confident are you that you can minimize the risk of bias in your own research? | **mean (SD):** 4.9 (1.2) [7 point scale] | | | | |

| **Introduction of bias into a trial:** | **Disagree**  **n (%)** | **Neutral**  **n (%)** | **Agree**  **n (%)** | **Don’t Know**  **n (%)** | **Total**  **n** |
| --- | --- | --- | --- | --- | --- |
| **Sequence Generation** | | | | | |
| Computer-generated randomization sequence | **141 (83.4)** | 8 (4.7) | 19 (11.2) | 1 (0.6) | 169 |
| Group allocation according to an alternating sequence | 48 (28.4) | 14 (8.3) | **101 (59.8)** | 6 (3.6) | 169 |
| Group allocation according to patient chart numbers | 36 (21.7) | 16 (9.6) | **108 (65.1)** | 6 (3.6) | 166 |
| Group allocation by flipping a coin | **86 (51.2)** | 25 (14.9) | 55 (32.7) | 2 (1.2) | 168 |
| **Allocation Concealment** | | | | | |
| Centralized group allocation (e.g., by the pharmacy or a call centre) | **88 (53.0)** | 18 (10.8) | 47 (28.3) | 13 (7.8) | 166 |
| **Blinding** | | | | | |
| Absence of blinding in a study with objective outcomes (e.g., mortality) | 49 (29.3) | 11 (6.6) | **105 (62.9)** | 2 (1.2) | 167 |
| Absence of blinding in a study with subjective outcomes (e.g., pain scale) | 20 (12.5) | 5 (3.1) | **133 (83.1)** | 2 (1.3) | 160 |
| **Selective Outcome Reporting** | | | | | |
| Reporting limited to statistically significant outcomes | 25 (15.0) | 16 (9.6) | **123 (73.7)** | 3 (1.8) | 167 |
| Reporting limited to clinically significant outcomes | 30 (17.9) | 17 (10.1) | **117 (69.6)** | 4 (2.4) | 168 |
| **Incomplete Outcome Reporting** | | | | | |
| Per-protocol analyses (analyzing participants’ results as treated, rather than according to original group assignments) | 24 (14.8) | 11 (6.8) | **121 (74.7)** | 6 (3.7) | 162 |
| Intention-to-treat analyses (analyzing participants’ results according to original group assignments, rather than as treated) | **102 (63.4)** | 14 (8.7) | 41 (25.5) | 4 (2.5) | 161 |
| Modified intention-to-treat analyses (analyzing results for participants who have met a set of minimum requirements) | *58 (36.0)* | 34 (21.1) | *58 (36.0)* | 11 (6.8) | 161 |
| **Other Sources of Bias** | | | | | |
| Trial stopped early for benefit | 40 (25.2) | 23 (14.5) | **90 (56.6)** | 6 (3.8) | 159 |
| Trial stopped early for harm | 50 (31.3) | 23 (14.4) | **81 (50.6)** | 6 (3.8) | 160 |
| Full industry sponsorship | 26 (16.3) | 21 (13.1) | **109 (68.1)** | 4 (2.5) | 160 |
| Partial industry sponsorship | 32 (20.0) | 25 (15.6) | **99 (61.9)** | 4 (2.5) | 160 |
| Receipt of industry donations (e.g., study drugs) | 48 (30.0) | 28 (17.5) | **81 (50.6)** | 3 (1.9) | 160 |

**Institution-level factors**

| **Item** | **Disagree**  **n (%)** | **Neutral**  **n (%)** | **Agree**  **n (%)** | **Don’t Know**  **n (%)** | **Total**  **n** |
| --- | --- | --- | --- | --- | --- |
| I find that applying methodologic research is often not practical in clinical trial settings | **89 (56.3)** | 18 (11.4) | 45 (28.5) | 6 (3.8) | 158 |
| I feel that I have access to sufficient resources (e.g., staff) to conduct a high-quality trial | 48 (30.4) | 16 (10.4) | **90 (58.4)** | - | 154 |
| I find that institutional requirements align well with efforts to minimize bias in conducting trials | 45 (29.2) | 20 (13.0) | **82 (53.2)** | 7 (4.5) | 154 |
| I find that the logistics of conducting a trial often make it difficult to minimize bias | 52 (33.5) | 19 (12.3) | **82 (52.9)** | 2 (1.3) | 155 |
| My colleagues conduct research that is methodologically rigorous | 12 (6.9) | 26 (14.9) | **131 (75.3)** | 5 (2.9) | 174 |
| I find it hard to report the details of my research as conducted because of space constraints in my target journals | 61 (39.6) | 17 (11.0) | **76 (49.4)** | - | 154 |
| I find it hard to publish my research without reporting statistically significant results | 33 (21.6) | 13 (8.5) | **102 (66.7)** | 5 (3.3) | 153 |
| I have the authority to change how research is conducted within my research group | 8 (5.1) | 7 (4.5) | **142 (90.4)** | - | 157 |
| I find that lack of sufficient funding limits how well I am able to conduct my research | 29 (18.7) | 17 (11.0) | **109 (70.3)** | - | 155 |
| I find that rigorous methods (e.g., adhering to standards such as those set out in the CONSORT Statement) are encouraged by one or more of my colleagues and/or supervisors | 9 (5.9) | 16 (10.5) | **123 (80.4)** | 5 (3.3) | 153 |
| I have opportunities to discuss research methods with knowledgeable colleagues | 8 (5.3) | 2 (1.3) | **141 (92.8)** | 1 (0.7) | 152 |
